# Supplementary material for: Binding to Iron Quercetin Complexes Increases the Antioxidant Capacity of the Major Birch Pollen Allergen Bet v 1 and Reduces Its Allergenicity
Source: Antioxidants (Basel). 2022 Dec 26;12(1):42. doi: 10.3390/antiox12010042 (PMC9854910; doi:10.3390/antiox12010042)
Supplement: Supplementary file 1 [file antioxidants-12-00042-s001.zip › antioxidants-2125487-supplementary.pdf]

Supplementary Information

Supplementary Figure S1

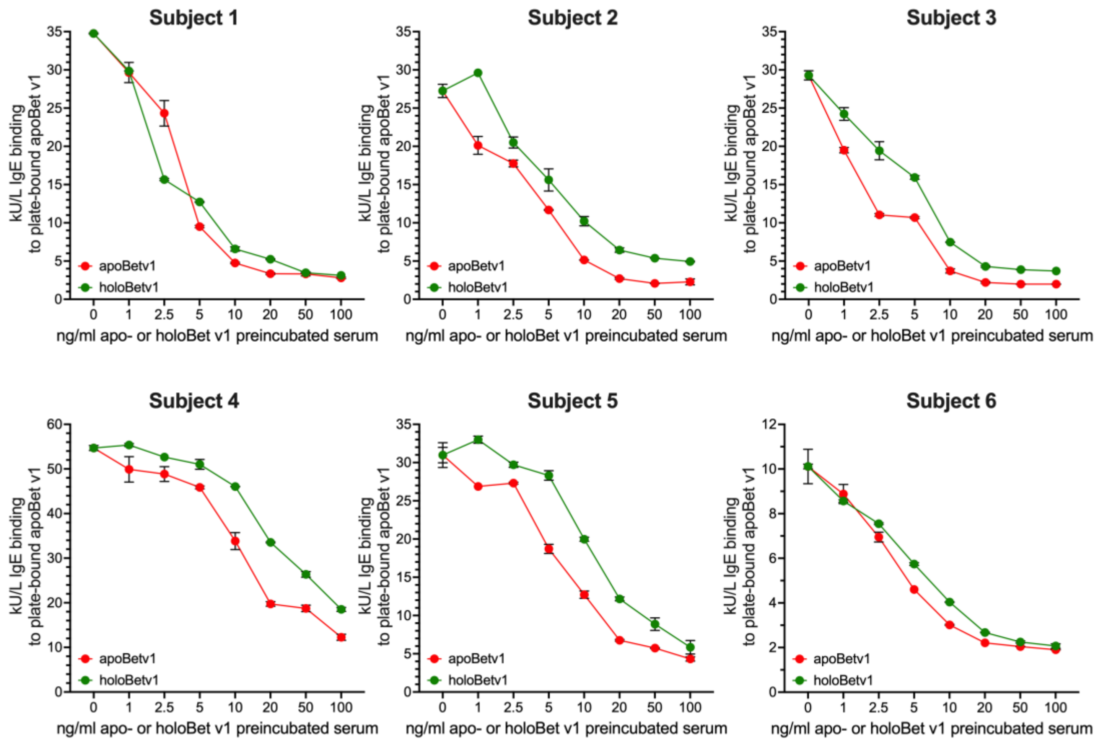

Supplementary Figure S1. Serum of 6 birch pollen allergic subjects were incubated with increasing concentration of apo- or holoBet v 1, before assessing the ability of the serum to bind to plate-bound apoBet v1.

Supplementary Figure S2

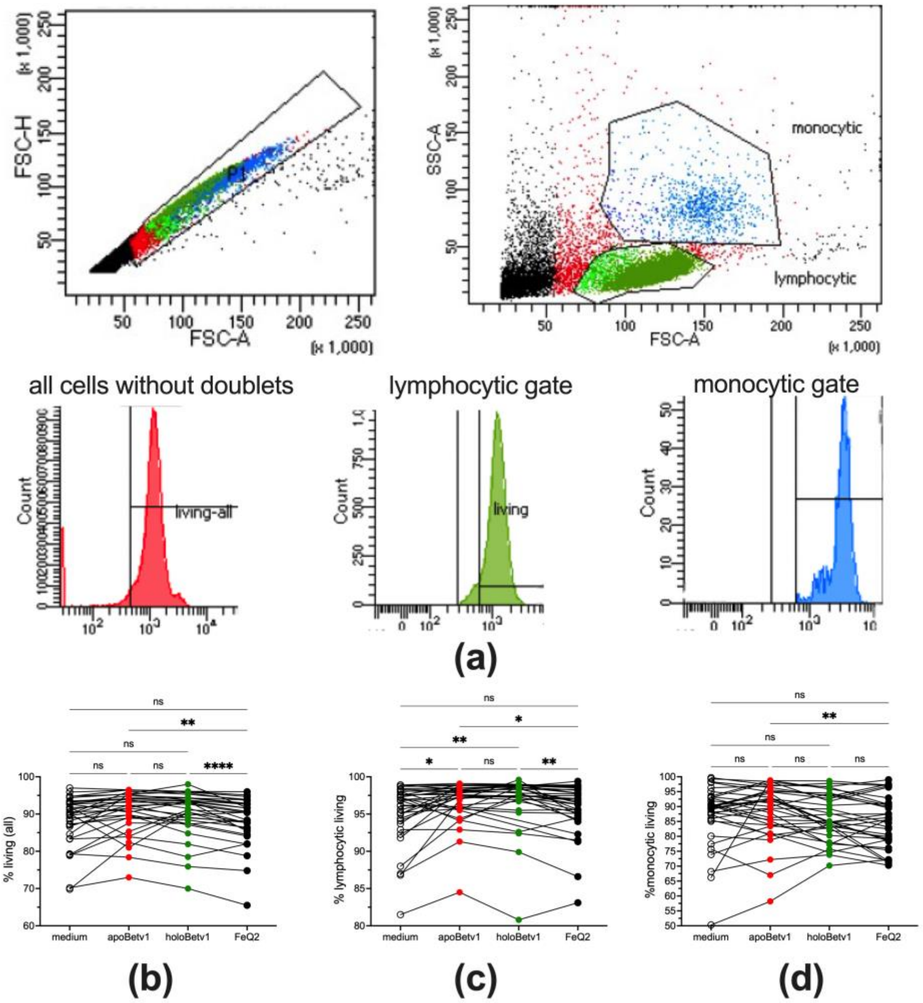

Supplementary Figure S2. No significant differences in the relative numbers of living cells in the gated cell population upon apo- or holoBet v1 stimulation. Peripheral blood mononuclear cells were incubated overnight in iron-free media alone or with quercetin, iron-quercetin in the presence or absence of apoBet v1. Cells were stained with calcein-AM and gated to the lymphocytic and monocytic compartment. Calcein positive cells within the gate were considered living. **(a):** Gating strategy, **(b):** relative living cell numbers of all gated cells, **(c):** relative living cell numbers in the lymphocytic gate **(d):** relative numbers of calcein-positve cells within the monocytic gate. Groups were compared by RM-one-way ANOVA following Tukey's multiple comparisons test. * $p<0.05$; ** $p<0.01$, ****$p<0.0001$.
